# Myopia prevalence and ocular biometry: a cross-sectional study among minority versus Han schoolchildren in Xinjiang Uygur autonomous region, China

**DOI:** 10.1038/s41433-021-01506-0

**Published:** 2021-08-19

**Authors:** Yumeng Shi, Yan Wang, Aizhi Cui, Sen Liu, Xiaolan He, Huijuan Qiu, Hanwen Cui, Yunxian Gao, Jin Yang

**Affiliations:** 1 Department of Ophthalmology and Visual Science, Eye Ear Nose and Throat Hospital of Fudan University, Shanghai, China; 2 NHC Key Laboratory of Myopia (Fudan University), Laboratory of Myopia, Chinese Academy of Medical Sciences, Shanghai, China; 3 Key Laboratory of Visual Impairment and Restoration of Shanghai, Shanghai, China; 4 Department of Ophthalmology, Traditional Chinese Medicine Hospital of Xinjiang Uyghur Autonomous Region, Ürümqi, Xinjiang China; 5 Department of Ophthalmology, Gaoyou Hospital Affiliated Soochow University, Gaoyou People’s Hospital, Gaoyou, Jiangsu China; 6 Department of Ophthalmology, Xinjiang Production and Construction Corps, Tacheng, Xinjiang China

**Keywords:** Epidemiology, Refractive errors

## Abstract

**Objectives:**

To describe and compare the prevalence of refractive error and its associated ocular biometric parameters in a large multi-racial sample of schoolchildren from Xinjiang.

**Methods:**

A total of 67,102 school children of five ethnicity groups aged 6–23 years from 46 schools in Xinjiang participated in this study. The children underwent a comprehensive eye examination for vision screening, including uncorrected visual acuity and standardized refraction. Refractive error was determined by autorefractors and subjective refraction. Refraction was recorded in spherical equivalent (SE). The age- and sex- adjusted prevalence of myopia (SE ≤ −0.5 D), low myopia (−6 D < SE ≤ −0.5 D), high myopia (SE ≤ −6.0 D), astigmatism (cylinder < −0.5 D), and anisometropia (difference in SE between two eyes of 1.0 D) in the five ethnic groups were calculated. Ocular biometric parameters including axial length (AL) and corneal radius of curvature (CR) were measured by AL-scan optical biometer.

**Results:**

The age- and sex- adjusted prevalence of myopia in the Han, Hui, Uyghur, Kyrgyz and Kazakh were 65.8% (95% confidence interval [CI] 65.4, 66.3); 59.1% (95% CI 57.8, 60.4); 30.1% (95% CI 29.2, 30.9); 30.2 (95% CI 28.9, 31.4); and 30.0% (95% CI 27.6, 32.3), respectively. The Han and Hui children also had longer ALs (Han, 23.8; Hui, 23.6, Uyghur, 23.1; Kyrgyz, 23.1; Kazakh, 23.3 mm) and larger AL/CR (Han, 3.04; Hui, 3.00; Uyghur, 2.95; Kyrgyz, 2.96; Kazakh, 2.97) values than the other three minorities (*P* < 0.01). Overall, girls had shorter ALs, steeper corneas, and smaller AL/CR values than boys (*P* < 0.01).

**Conclusions:**

Significant ethnic difference in the prevalence of myopia was observed in this study on school-aged children in Xinjiang (Han > Hui > Kyrgyz > Uyghur > Kazakh). This study among different ethnic groups in a multiethnic population is valuable for enriching the ethnical information resources for refractive errors and ocular biometry parameters, as well as facilitating further research on myopia-related diseases and risks.

## Introduction

Based on the growing prevalence of myopia around the world, particularly in the younger generations in East Asia, myopia has emerged as a major health issue causing significant visual loss and is also a risk factor for a range of other serious ocular pathologies [1, 2]. Multiethnic population-based studies have identified wide interethnic variations in the prevalence of myopia among different ethnic groups. For example, the prevalence of myopia among East Asians is over twice as high as similarly aged Caucasian (white) persons [3]. Moreover, the age-matched prevalence of myopia is higher in people of Chinese ethnicity compared with other ethnic groups in Singapore, a city-state with one of the highest prevalence rates of myopia in the world [4–6]. Nevertheless, studies on migrant populations have shed new light, including studies on students of Chinese origin in Australia, yet showing lower levels of myopia than those in urban centers in east and southeast Asia [1, 7]. Therefore, there is still a clear necessity to address the issue of whether the environments to which people are exposed or genetic ancestry, account mainly for the significant interethnic disparities pertaining to the prevalence of myopia. Xinjiang Uygur autonomous region, as a major population center for ethnic minorities in China, is a perfect region to investigate the ethic disparities due to its unique geographic and demographic reasons.

Recently, the epidemiology of myopia in school-aged children has been well established with a large number of population-based studies in different parts of China [8–11]. It was once reported that up to 90% of Chinese teenagers and young adults were short-sighted [12]. However, the study participants were predominantly of Han ethnicity, and there is a paucity of data on the prevalence of myopia of other ethnic groups in China.

Until now, there has still been a lack of detailed and systematic studies on the prevalence of myopia and its related ocular biometry in school-aged children in Xinjiang. Findings from this study may help fill the gap in knowledge about ethnic minorities and probably lead to a further step towards a comprehensive children vision screening system in China, in line with the national children’s myopia management plan [13].

In this article, we describe and compare the prevalence of refractive errors and ocular dimensions in school-based samples of Han, Uyghur, Hui, Kyrgyz, and Kazakhs people living in Xinjiang and discuss the possible risk factors accounting for ethnic differences in prevalence.

## Methods

### Study population

The Xinjiang minority eye study was a cross-sectional, school-based study that was performed in Xinjiang Uygur autonomous region. With China’s longest land border, Xinjiang is inhabited by more than 40 different ethnic groups. This school-based prevalence study of myopia and ocular parameters in five areas of Xinjiang (Ürümqi, Kashi Prefecture, Tacheng Prefecture, Ili Kazakh Autonomous Prefecture, and Kizilsu Kirghiz Autonomous Prefecture), was conducted from May 2019, and recruitment and data collection are ongoing (currently *N* = 67,102). The stratified random cluster sampling strategy was devised for this study based on schools in the relevant geographical location. Ürümqi, Tacheng, and Ili are located in Northern Xinjiang while Kashi and Kizilsu, in Southern Xinjiang. Overall, students from 17 primary schools (students aged 6–11), 20 junior high (students aged 12–14), and 9 senior high schools (students aged 15 or over) were recruited. This study was part of a national children’s myopia management plan, which has focused on the screening and prevention of adolescent myopia and the establishment of comprehensive archives for eye development, thus the participation rates in each ethnic group were extremely high. Informed verbal consent was obtained from the students and/or the guardians of the children, and all subjects were treated in accordance with the tenets of the Declaration of Helsinki.

### Ocular examinations

Ocular examinations including the measurement of visual acuity and refraction were performed by a trained team of ophthalmologists and optometrists from Shanghai and Xinjiang. Repeated review and testing of the quality of all technicians’ performances were conducted. Cycloplegia was induced with three drops of 1% cyclopentolate instilled 5 min apart, and pupillary dilation of at least 6 mm with the absence of light reflex was considered complete cycloplegia. Autorefraction measurements in the left and right eye were performed using one of two autorefractors (ARK-1, AR-1, NIDEK, Tokyo, Japan), and the average of refractive error readings was taken. Refinement of the sphere, cylinder, and axis was performed until the best VA was obtained. Final refraction was determined using subjective refraction by the trained team. Ocular biometric parameters including AL and CR in the horizontal and vertical meridian were measured using an AL-Scan Optical Biometer (AL-scan, NIDEK, Tokyo, Japan) as the average of three recordings, and CR was calculated as the mean of the longest CR and shortest CR. The axial length-to-corneal radius (AL/CR) ratio was defined as the AL divided by the mean CR.

### Definitions

The spherical equivalent (SE) of the refractive error, defined as the spherical value of refractive error plus one-half of the cylindrical value, was used to classify participants as myopic, low myopic, and high myopic. Myopia was defined as a condition in which the SE ≤ −0.5 D in either eye. We standardized to a SE of −6.0 D or less in either eye for high myopia according to the international myopia institute myopia control reports in 2019 [14]. SE ≤ −0.5 and > −6.0 D was termed low myopia. Astigmatism analyzed in minus cylinders was defined as less than −0.5 D of cylinder and anisometropia was defined as the difference in SE between the right and left eyes of 1.0 D.

### Data analysis

As the Spearman correlation coefficients for SE (*r* = 0.845) and ocular biometric parameters in the left and right eye were high (*r* = 0.959 for AL; *r* = 0.960 for CR), and the results of the analysis in both eyes were similar, only right eye data are presented. Prevalence was calculated as the number of participants with the particular type of refractive error in relation to the total number of subjects examined by age and ethnic group, and is given as mean ± standard error; 95% confidence intervals (95% CI) are also included. Both the crude and age-sex-standardized prevalence of the refractive errors are presented. The chi-square test was used to compare the prevalence difference between the five ethnic groups. To compare the prevalence of refractive error between age, sex, and ethnicity (Han versus minorities), a logistic regression analysis was applied, and odds ratios (OR) and their 95% CI are presented. A linear regression analysis was applied for continuous variables to examine the associations between age, sex, ethnicity, and ocular biometric parameters, with coefficient *β* and their 95% CI presented. Statistical analyses were carried out using SPSS for Windows (version 24.0; IBM-SPSS, Chicago, IL, USA). A *P* value of 0.05 or less was considered statistically significant.

## Results

With similar participation rates among the five ethnic groups (Han, 95.9%; Hui, 98.8%, Uyghur, 91.7%; Kyrgyz, 95.2%; Kazakh, 92.2%), the study eventually included 67,102 students (33989 [50.7%] boys) with a mean age of 12.7 ± 3.3 years (median: 12.4 years; range, 6–23 years). In terms of ethnicity, 43,915 (65.4%) students were Han, and 11,398 (17.0%), 5268 (7.9%), 5056 (7.5%), and 1465 (2.2%) were Uyghur, Hui, Kyrgyz, and Kazakhs, respectively (Table 1).

**Table 1:** Demographic characteristics of 67,102 participants among the five ethnic groups.

	Han *N* = 43,915		Uyghur *N* = 11,398		Hui *N* = 5268		Kyrgyz *N* = 5056		Kazakhs *N* = 1465	
	*n*	%	*n*	%	*n*	%	*n*	%	*n*	%
Age, year (SD)	12.30	3.01	13.88	4.02	12.08	2.89	14.07	3.52	13.26	3.36
*Age, year*										
6–12	21,904	49.90%	4133	36.30%	2748	52.20%	1542	30.50%	555	37.90%
12–18	19,921	45.40%	4905	43.00%	2352	44.60%	2800	55.40%	775	52.90%
>18	2090	4.80%	2360	20.70%	168	3.20%	714	14.10%	135	9.20%
*Sex*										
Male	22,857	52.00%	5169	45.40%	2707	51.40%	2513	49.70%	743	50.70%
Female	21,058	48.00%	6229	54.60%	2561	48.60%	2543	50.30%	722	49.30%

Data presented are means (SD: standard deviations) or number (%), as an appropriate variable.

**Fig. 1:**
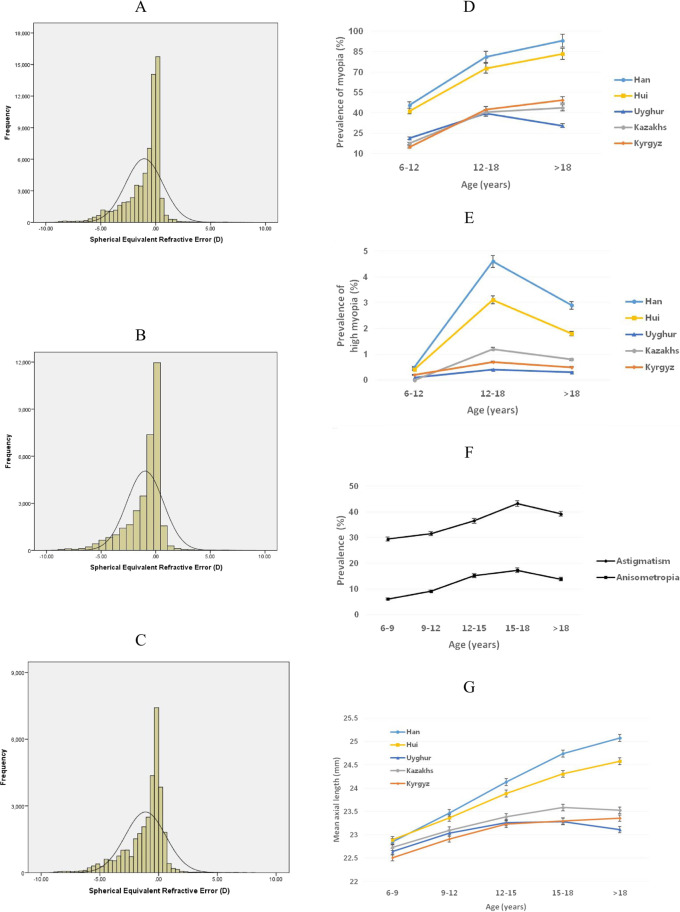
The distribution and prevalence of refractive errors in the Xinjiang eye study. **A** Distribution of refractive errors in diopters for all subjects. **B** Distribution of refractive errors in diopters for all male subjects. **C** Distribution of refractive errors in diopters for all female subjects. **D** Age-specific prevalence of myopia (SE ≤ −0.5 D) in five ethnic groups. **E** Age-specific prevalence of high myopia (SE ≤ −6.0 D) in five ethnic groups. **F** Age-specific prevalence of astigmatism (cylinder < −0.5 D) and anisometropia (difference in SE between two eyes of 1.0 D) for all subjects. **G** Mean axial length by age in five ethnic groups.

The respective mean SE values for all subjects, male and female subjects were −1.0 ± 1.7 D (median, −0.4; range, −18.0 to +11.9 D), −1.0 ± 1.7 D (median, −0.4; range, −18.0 to +11.9 D), and −1.1 ± 1.7 D (median, −0.5; range, −17.4 to +8.1 D; Fig. 1A–C); male subjects demonstrated a significantly less myopic mean SE than female subjects (*P* < 0.001). Overall, the prevalence of myopia was 54.9%. The median (range) SE was −0.8 D (−18 to 8.6 D) in the Han students, −0.50 D (−12.8 to 8.0 D) in the Hui, −0.1 (−9.3 to 11.9) in the Uyghur, −0.1 D (−7.5 to −6.5 D) in the Kazakhs, and −0.1 D (−8.5 to 7.9 D) in the Kazakhs.

Table 2 describes the crude and age-sex-standardized prevalence of myopia (SE ≤ −0.5 D), high myopia (SE ≤ −6.0 D), astigmatism (cylinder < −0.5 D), and anisometropia (difference in SE between two eyes of 1.0 D) between the five ethnic groups. The ethnicity patterns of myopia showed a high prevalence in students of Han (65.9%) and Hui (59.1%) and relatively low in students of Uyghur (30.1%), Kyrgyz (30.2%), and Kazakhs (30.0%). Similar ethnic patterns were found in the prevalence of low myopia, high myopia, and anisometropia. In terms of astigmatism, students of Uyghur (25.5%) had a lower prevalence of astigmatism than their counterparts of the other four ethnicity groups. The age-specific prevalence of myopia was 45.6%, 81.2%, and 93.0% in students of Han aged 6–12, 12–18, and older than 18 years and it was 41.2%, 72.7%, and 83.3% in students of Hui for the same age groups. The prevalence of myopia increased with age groups in all ethnic groups except the Uyghur (21.2%, 39.4%, and 30.5%), whereas the age-specific prevalence of high myopia is highest in the 12–16 age group in all ethnic groups (Fig. 1D, E). For both astigmatism and anisometropia, there was a monotonic increase in prevalence with age groups (Fig. 1F).

**Table 2:** Prevalence of refractive errors by an ethnic group with age and sex-adjusted rates.

Ethnicity	Myopia (≤−0.5D)		Low myopia (−6D to 0.5D)		High myopia (≤−6D)		Astigmatism (<−0.5 cylinder)		Anisometropia	
	*n*	%; 95% CI	*n*	%; 95% CI	*n*	%; 95% CI	*n*	%; 95% CI	*n*	%; 95% CI
Han (*N* = 43,915)	28100		26,842		1258		16,875		6480	
Crude		63.99; 63.54–64.44		61.12; 60.66–61.58		2.86; 2.71–3.02		38.43; 37.97–38.88		14.76; 14.43–15.09
Age and sex standardized		65.84; 65.39–66.28		62.59; 62.14–63.04		3.25; 3.08–3.41		39.14; 38.69–39.60		15.50; 15.16–15.84
Uyghur (*N* = 11,398)	3531		3495		36		2930		652	
Crude		30.98; 30.13–31.84		30.66; 29.82–31.52		0.32; 0.22–0.44		25.71; 24.91–26.52		5.72; 5.30–6.16
Age and sex standardized		30.05; 29.20–30.89		29.77; 28.93–30.61		0.28; 0.18–0.38		25.45; 24.65–26.25		5.92; 5.49–6.36
Hui (*N* = 5268)	2982		2888		94		1734		681	
Crude		56.61; 55.25–57.95		54.82; 53.47–56.17		1.78; 1.44–2.18		32.92; 31.65–34.20		12.93; 12.03–13.86
Age and sex standardized		59.09; 57.76–60.42		56.96; 55.62–58.29		2.13; 1.74–2.52		34.02; 32.74–35.30		13.51; 12.58–14.43
Kyrgyz (*N* = 5056)	1765		1739		26		1640		240	
Crude Age and sex standardized		34.91; 33.59–36.24 30.15; 28.88–31.41		34.39; 33.08–35.72 29.70; 28.44–30.96		0.51; 0.34–0.75 0.45; 0.26–0.63		32.44; 31.15–33.75 32.37; 31.08–33.66		4.75; 4.18–5.37 4.44; 3.87–5.01
Kazakhs (*N* = 1465)	468		456		12		500		108	
Crude		31.95; 29.56–34.40		31.13; 28.76–33.57		0.82; 0.42–1.43		34.13; 31.70–36.62		7.37; 6.09–8.83
Age and sex standardized		29.97; 27.63–32.32		29.26; 26.93–31.59		0.71; 0.28–1.14		33.79; 31.37–36.21		7.04; 5.73–8.35
P*		<0.01		<0.01		<0.01		<0.01		<0.01

Adjusted for age and sex.

Data are presented as value; 95% confidence interval.

*P values are based on age- and the sex-adjusted difference in means between the five ethnic groups.

In multivariate logistic regression analysis, with adjustment for age and sex, the presence of the degree of myopia and anisometropia was associated with older age (all *P* < 0.01) and female sex (all *P* < 0.01). However, the presence of astigmatism was associated with older age (*P* < 0.01) and male sex (*P* < 0.01). Compared with the Uyghurs, those of Hui origin were around six times more likely to be myopic, whereas the Han children were around eight times more likely to be myopic. The Han students also had higher OR of low myopia (6.2), high myopia (21.4), astigmatism (2.1) as compared with the Uyghurs, and anisometropia (4.6) as compared with the Kyrgyz (Table 3).

**Table 3:** Multivariate analysis of the associations between refractive errors, ocular biometry, and ethnicity.

	Myopia (≤−0.5D)	Low myopia (−6D to 0.5D)	High myopia (≤−6D)	Astigmatism (<−0.5 cylinder)	Anisometropia	AL (mm)	CR (mm)	AL/CR
	Adjusted OR (95%CI)	Adjusted OR (95%CI)	Adjusted OR (95%CI)	Adjusted OR (95%CI)	Adjusted OR (95%CI)	Adjusted β (95%CI)	Adjusted β (95%CI)	Adjusted β (95%CI)
*Age*	1.29 (1.29–1.30) #	1.24 (1.23–1.24) #	1.40 (1.38–1.43) #	1.08 (1.07–1.08) #	1.15 (1.14–1.15) #	0.16 (0.15, 0.16) #	0.000121 (0.000, 0.001)	0.02 (0.02, 0.02) #
*Sex*								
Male	Reference	Reference	Reference	Reference	Reference	Reference	Reference	Reference
Female	1.31 (1.27–1.35) #	1.28 (1.23–1.32) #	1.17 (1.05–1.31) #	0.87 (0.84–0.89) #	1.21 (1.15–1.27) #	−0.43 (−0.45, −0.42) #	−0.12 (−0.12, −0.11) #	−0.01 (−0.01, −0.01) #
*Ethnicity*								
Han	8.12 (7.69–8.56) #	6.15 (5.84–6.47) #	21.40 (15.28–29.97) #	2.05 (1.95–2.15) #	4.63 (4.05–5.30) #	0.98 (0.95, 1.01) #	0.04 (0.04, 0.05) #	0.11 (0.10, 0.11) #
Uyghur	Reference	Reference	Reference	Reference	1.20 (1.03–1.40)*	0.01 (−0.02, 0.04)	0.04 (0.03, 0.05) #	−0.01 (−0.02, −0.01) #
Hui	6.01 (5.57–6.48) #	4.82 (4.48–5.19) #	14.42 (9.74–21.33) #	1.63 (1.52–1.76) #	4.09 (3.50–4.77) #	0.78 (0.75, 0.82) #	0.06 (0.05, 0.07) #	0.08 (0.07, 0.08) #
Kyrgyz	1.25 (1.15–1.34) #	1.22 (1.13–1.31) #	1.84 (1.11–3.05)*	1.37 (1.28–1.48) #	Reference	Reference	Reference	Reference
Kazakhs	1.34 (1.18–1.52) #	1.24 (1.09–1.40) #	3.97 (2.05–7.67) #	1.58 (1.40–1.78) #	1.81 (1.43–2.29) #	0.28 (0.22, 0.34) #	0.03 (0.01, 0.04) #	0.02 (0.02, 0.03) #

Adjusted for age and sex.

*OR* odds ratio, *AL* axial length, *CR* corneal curvature of radius, *CI* confidence interval.

*P < 0.05, #*P* < 0.01.

After adjusting for the effect of age and sex (the crude not presented), the Han students aged 6–12, 12–18, and older than 18 years had 0.4 mm, 1.0 mm, and 1.9 mm longer in AL on average respectively compared with their counterparts of Uyghurs while the Hui students had 0.3 mm, 0.7 mm and 1.5 mm longer in AL for the same age groups compared with Uyghurs on average. Marked increases in the mean AL with age were shown in all the ethnicity groups except in Uyghurs (Fig. 1G). The mean CR and AL/CR were slightly longer in people of Han and Hui ethnicity compared with those of the other three ethnicity students (Table 4).

**Table 4:** Ocular biometry adjusted for age and sex among the five ethnic groups.

Ethnicity	AL (mm)		CR (mm)		AL/CR	
	Mean	95% CI	Mean	95% CI	Mean	95% CI
Han (*N* = 43,915)						
6–12 years	23.26	23.25, 23.28	7.85	7.85, 7.85	2.97	2.96, 2.97
12–18 years 18+ years	24.32 25.06	24.31, 24.34 25.01, 25.12	7.85 7.87	7.85, 7.86 7.86, 7.88	3.10 3.19	3.10, 3.10 3.18, 3.19
Uyghur (*N* = 11,398)						
6–12 years	22.87	22.85, 22.89	7.86	7.85, 7.87	2.91	2.91, 2.91
12–18 years 18+ years	23.28 23.15	23.26, 23.31 23.12, 23.19	7.84 7.82	7.84, 7.85 7.81, 7.83	2.97 2.96	2.97, 2.97 2.96, 2.97
Hui (*N* = 5268)						
6–12 years	23.20	23.17, 23.24	7.87	7.86, 7.88	2.95	2.95, 2.95
12–18 years 18+ years	24.00 24.61	23.96, 24.05 24.40, 24.82	7.86 7.88	7.85, 7.87 7.84, 7.92	3.05 3.12	3.05, 3.06 3.10, 3.15
Kyrgyz (*N* = 5056)						
6–12 years	22.78	22.74, 22.82	7.82	7.81, 7.84	2.91	2.91, 2.92
12–18 years 18+ years	23.26 23.38	23.23, 23.29 23.31, 23.45	7.80 7.81	7.79, 7.81 7.79, 7.83	2.98 3.00	2.98, 2.99 2.99, 3.00
Kazakhs (*N* = 1465)						
6–12 years	22.95	22.88, 23.02	7.86	7.83, 7.88	2.92	2.92, 2.93
12–18 years 18+ years	23.47 23.57	23.41, 23.54 23.38, 23.75	7.83 7.82	7.81, 7.85 7.77, 7.87	3.00 3.02	2.99, 3.01 3.00, 3.04

Adjusted for age and sex.

Data presented are means (standard deviations).

*AL* axial length, *CR* corneal curvature of radius, *CI* confidence interval.

In linear regression analysis, older age was associated with AL and AL/CR (both *P* < 0.01), but not CR (*P* = 0.69). The girls had shorter ALs (by 0.4 mm), a steeper cornea (by 0.1 mm), and lower AL/CR (by 0.01). All the ethnicity groups except the Uyghur were associated with AL, whereas all of them (all *P* < 0.01) were associated with CR and AL/CR (Table 3).

### Effect of adjusting ethnic differences in the prevalence of myopia for ocular dimensions

Ethnic variations in myopia prevalence (adjusted for age and sex) were weakened after further adjustment for AL. The OR (95% CI) comparing the Han with the Uyghurs was reduced by a large amount from 8.1 (7.7–8.6) to 4.1 (3.8–4.3) after adjustment for AL. Further adjustment for CR and AL/CR weakened the OR further to 2.9 (2.7–3.1). In the comparison of the Hui students with the Uyghurs, after adjustment for AL, the OR was reduced from 6.0 (5.6–6.5) to 3.3 (3.0–3.6) and to 2.7 (2.5–2.9) after additional adjustment for CR and AL/CR. Ethnic variations in mean SE were also weakened after adjustment for AL, CR, and AL/CR but remained statistically significant. Compared with the Uyghurs, the Han and Hui had a more myopic SE of −0.1 D (−0.1 to −0.8) and −0.1 D (−0.2 to −0.1 D), respectively, after full adjustment, with the largest contribution being ethnic differences in AL. Adjustment for AL reduced the total residual variance in SE by 43%.

## Discussion

This study among elementary, middle, and high school students of five various ethnicity groups residing in Xinjiang provided novel data for specific comparison of ethnic disparities in the prevalence of myopia or degree of myopia and ocular measurements. This study indicated that the Han students had a higher prevalence of myopia and higher levels of negative astigmatism than students of other ethnicity groups, and the Hui students also showed a higher prevalence of myopia compared with the Uyghur, Kazakhs, and Kyrgyz, but not to the same degree as the Han. Overall, the age- and sex-adjusted prevalence of myopia was 65.8%, 59.1%, 30.2%, 30.1%, and 30.0% in Han, Hui, Kyrgyz, Uyghur, and Kazakhs participants, respectively. The adjusted prevalence of high myopia was 3.3%, 2.1%, 0.5%, 0.3%, and 0.7% in those five ethnicity groups respectively. Differences in myopia prevalence and SE corresponded with ethnic variations in ocular parameters, with the Han and the Hui students having longer ALs and higher AL/CR values.

The significant ethnic variations obtained in this study are comparable with several other multiethnic comparison studies in China. In a cross-sectional study of 10,037 students aged 9–12-year-old from Yunnan and Guangdong, subjects of the Yunnan minorities were significantly less myopic than those of Han ethnicity [15]. One study among Han and Yugur adults in Northwest China showed that myopia was more common among Han adults [16]. Another similar study conducted among Han and Yi adults in Southwest China had come to the same conclusion [5]. As for the Xinjiang minorities investigated by few studies, there was one on 646 children from five schools aged 4–19 years conducted in a rural area, Turpan, reporting a higher myopia prevalence of the Han (27%) than those of the Hui (18%) and the Uyghur (13%) [17]. Possible explanations for this result of an overall lower prevalence than ours are that our study had a larger sample size with a narrower age range and most of our randomly-selected schools are in the city areas.

Although the reasons for ethnic disparities in myopia prevalence between Han and non-Han children are not well understood, there are some aspects that may account for this. Myopia is a consequence of combinational effects of multiple genetic factors and indoor and outdoor environmental exposures. It is interesting to note that some ethnic minority groups of China including the Hui harbor a genetically close relationship with the Han majority, while significant genetic differences do exist between the Han and other minority groups, most prominently for the Kazakh, Kyrgyz, and the Uyghurs, etc. [18–20]. Compared with the other three studied minority groups, the Hui communities are more scattered all across China instead of merely concentrated in some specific provinces. Given a more frequent Han–Hui intermarriage and the same widely used language, Mandarin, we can assume that the amalgamation and assimilation among Han and Hui are far more common than other minorities [21]. Therefore, our findings that myopia prevalence is higher among the Hui and Han populations, as well as their longer AL, may be partially explained by the relatively close genetic relationships between these two ethnic groups. However, the Uyghurs, as the main ethnic groups in Xinjiang, present a typical mixture of Western and Eastern anthropometric features [22]. Shuhua Xu reported that 60% European ancestry and 40% Asian ancestry constituted the Uyghur population through analyzing their genomic admixture, and such admixture occurred approximately 126 generations ago [22]. In addition, phylogenetic analysis indicates that Kyrgyz and Kazakh people are genetically close to Uyghurs [23, 24]. Hence, it is reasonable to infer that these three Turkic-speaking minorities may have a less genetic predisposition to myopia compared with the Han nationality. Research also shows that the Han populations present a high genetic homogeneity all over China [20], which can explain our findings of the same high level of myopia prevalence in the Han children even in the frontier areas of China as those dwelling in other parts of the country. (see below for further discussion)

Although we can assume the environments that the children are exposed to and the schooling system share a high degree of similarity because our random sampling strategy is school-based conducting in the same area, each studied ethnic group yet has its unique culture and specific lifestyle. Generally, the Uyghur, Kyrgyz and Kazakh people are more nomadic and living in a more dispersed manner, while the Han culture stresses the importance of children’s academic achievements and earlier education, indicating that children of Han ethnicity might devote more time to reading indoors and less time to exercising outdoors, and the Hui parents are more like the Han instead of others. In addition, the Han children attend school at an earlier age than their non-Han counterparts, resulting in a relatively moderate upward trend of AL with age in minority children in our study. Even a slightly reverse relationship between increasing AL and older age was observed when the intrinsic growth of eyeball outweighed the external factors, especially for the Uyghur children due to being about 1–2 years older.

Another finding of this study is that even in the northwest border of China, myopia prevalence among the Han schoolchildren is high. On the whole, this school-based study confirmed previous findings indicating that the trend towards a higher prevalence of myopia in the younger generation of the Han ethnicity in China. The prevalence of myopia in Han students in our study was 45.6%, 81.2%, and 93.0% in the age group 6–12 years, 12–18 years, and older than 18 years, respectively. Based on a large sample size of the Han Chinese (*n* = 43,915) living in minority areas, the results of this study in Han can be compared with previous ones conducted in Eastern China where mostly Han Chinese reside. In 2012, Zhou and coworkers published the results of a school-based epidemiological study in 6 provinces in China with an overall prevalence of myopia of 55.7%, and myopia was present in 35.8%, 58.9%, 73.4%, and 81.2% of students aged 6 to 8, 10–12, 13–15 and 16–18 years old [25]. In 2013, a cohort study in Shanghai revealed that myopia increased by 16.0% at one-year follow-up among 4814 primary students aged 6 to 10 years [26]. Our findings of a high prevalence of myopia in senior students agree with a retrospective study carried out in Fenghua, Eastern China on 43,858 students aged 18.46 ± 0.69 years in their third year of high school. From 2001 to 2015, the increase of the prevalence of myopia was from 79.5% to 87.7% [27]. However, this increasing trend mentioned above was more obvious in recent findings. Examining 14,551 aged 5–16 years from 42 primary schools and 17 middle schools in Tianjin in the year 2018, myopia was found in 78.2% of the children [28].

For the Han Chinese living in the western areas, our result is similar to the investigation in Western China conducted by Guo et al. [11] in 2014, which showed that the overall prevalence of myopia in children aged 6–21 years was 60.0 ± 1.2%. With the same diagnostic threshold for high myopia (SE ≤ −6 D) as ours, the overall prevalence of it is slightly lower than our results (2.9% vs. 3.3%). Nevertheless, their findings of an increasing tendency with the grade in high myopia prevalence seem inconsistent with ours. On this point, the highest rate occurred in the age group 12–18 years (4.6%) in our participants. One reason could be the relatively smaller proportion of participants in age >18 than those between 12 and 18 years old in our study.

In the present study, the observed gender differences in myopia prevalence (females vs. males, OR 1.3; 95% CI, 1.3–1.4; *P* < 0.01) was also supported by other similar studies, with higher levels of myopia in girls [10, 29–31]. One possible explanation is that girls tend to spend more time near work rather than outdoor sports. Another sex-related observation that the boys have a longer AL and CR than the girls are in agreement with others [30–32]. We also found that boys have a higher ratio of AL to CR, which is a better marker of myopia progression compared with AL [33]. The phenomenon that the anisometropia prevalence increase with age in this study was also reported by previous research, indicating that it is strongly related with age yet showing a slowdown in progress later in childhood [28, 34, 35]. Furthermore, a similar pattern was found in the prevalence of astigmatism as a whole.

The strengths of the study included a multiethnic, large school-based sample, standardized refraction, and ocular biometry measurements. Data collected was based on the same methodology and by the same ophthalmologists and technicians, to ensure the validity of interethnic comparison. Despite the study’s strengths, several potential limitations should be noted. First, we were unable to ensure similar numbers of children of different ethnicity groups because our random sampling strategy is school-based, however, the high and similar response rates, as well as the large sample size of each ethnic group, help to limit the role of selection bias. Second, risk factors assessment in association with myopia was absent in the present study because the large sample size made it difficult to carry out a questionnaire survey. Third, we did not measure height, which would have complemented our data for multiple regression analysis. Notwithstanding these limitations, this study provided novel data for myopia prevalence and ocular dimensions among different ethnic groups in the same geographic region of China.

In conclusion, this study of multiethnic schoolchildren in the border province of China found significant ethnic disparities in myopia prevalence and its related ocular biometric parameters, with Han and Hui Chinese having a higher prevalence of myopia, high myopia as well as longer ALs and larger AL/CR values as compared with the Uyghur, Kyrgyz and Kazakh minorities. Further well-designed cohort studies are warranted to confirm the exact factors explaining the observed ethnic variations, which is conducive to formulating myopia control strategies for China and other countries.

### Summary

#### What was known before

- Multiethnic population-based studies have identified wide interethnic variations in the prevalence of myopia among different ethnic groups. Myopia prevalence among the Han schoolchildren in Eastern China is high and has been increasing.

#### What this study adds

- This school-based study on myopia and ocular parameters in 67102 students in Xinjiang Uygur autonomous region indicated that the Han and the Hui students with longer ALs and higher AL/CR values had a comparatively higher prevalence of myopia than students of other ethnicity groups. Even in the northwest border of China, myopia prevalence among the Han schoolchildren is high.
